# Compliance with clinical guidelines: the role of incentives and competition between practitioners

**DOI:** 10.1007/s10198-025-01784-5

**Published:** 2025-04-28

**Authors:** Gianluca Fiorentini, Luke B. Connelly

**Affiliations:** 1 Department of Economics, The University of Bologna, 40126 Bologna, BO Italy; 2 Centre for the Business and Economics of Health, The University of Queensland, Brisbane, 4072 Australia; 3 Department of Sociology and Business Law, The University of Bologna, 40126 Bologna, BO Italy

**Keywords:** Multiple principals, Compliance with clinical guidelines, Chronic disease management programs, GPs/specialists' competition, Incentives, C23, D22, I11, I18, L10

## Abstract

In this study we use a unit record, panel dataset, to examine the behaviour of clinicians under a chronic disease management program (CDMP) that is designed to improve care and slow the progression of chronic kidney disease (CKD). Using 8 years of quarterly data on the population of CKD patients (*n* = 44,686) in the Emilia-Romagna region of northern Italy, we analyse a setting where medical practitioners are agents who respond to the demands of two principals: their patient and the third-party payer. Exploiting detailed information on specialist visits and the concentration of general practitioners (GP) in local areas, as well as disease severity, we study how GPs on one side, and specialist nephrologists and cardiologists on the other comply with the CDMP guidelines, as may be predicted on the basis of a dual-agency approach. Our application of both multiple-treatment differences-in-differences (*DID*_*M*_) and panel fixed-effects linear probability models produces evidence coherent with income-maximizing and, to a lower extent, effort-reducing strategies on the part of the GPs, as well as with strategic behaviour by some specialists. We also produce evidence that specialists who practice in hub facilities with a leading role in CDMP implementation, also for reputational reasons, exhibit referral practices that are more closely related to the CDMP guidelines.

**Supplementary Information:**

The online version contains supplementary material available at 10.1007/s10198-025-01784-5.

## Introduction

Chronic Disease Management Programs (CDMPs) (or “Integrated Care Programs” (ICPs) have received considerable attention over the past 20 years, both in the clinical and applied health economics literatures. One reason for this is that the burden of chronic disease (CD) has grown as life-expectancy has increased. According to the most recent Global Burden of Disease (GBD) study, for instance, chronic (or “non-communicable”) diseases now account for 97% of all deaths and 87% of years lived with disability in Europe [11]. The promise that CDMPs could produce substantial improvements in these outcomes has, however, scantly been realised. For instance, Bleich et al. [2] conducted a systematic review of 27 studies of programs to treat people with multi-morbid CD, focusing on intended effects that included improvements in the efficiency of health care use, reductions of health expenditure, improvements in clinical outcomes or improvements in patient satisfaction. The authors reported that many studies reported no significant change on any of these aims and remarked that this was “…an especially surprising result given the expected publication bias of reporting only favourable results” (p.197).

Understanding the reasons that some CDMP programs fail to deliver their objectives is an important endeavour. Given the contemporary emphasis on, and allocation of substantial resources to CDs, economists have an important role to play in understanding drivers of the success and failure of such programs (Connelly, Fiorentini and Iommi [23]). CDMPs typically involve attempts to improve the care provided to people with specific chronic diseases by specifying protocols, or guidelines, for their treatment. Those guidelines are usually staged according to the severity of disease—which are often conceived as representing different risk classes—with different treatment regimens recommended for those disease stages, with a view to improving the care and outcomes for people. Much of the literature (see, e.g., Connelly and Fiorentini [23], for a review) on this topic seeks to identify the reasons that patients do not adhere to CDMP recommendations. An under-researched question is whether or not medical practitioners, themselves, face sufficient incentives to implement the recommended treatment regimens inherent in CDMPs.

The vast literature on the determinants of adherence to clinical guidelines and the (in)effectiveness of some CDMPs presents a number of hypotheses as to why CDMPs sometimes fail to live up to expectations. For instance, many of these works focus on the role of demand-related factors [6, 20, 21] such as the effective provision of information to patients, the cost of getting access to services, or the costs to patients of compliance with the guidelines. While such demand-side factors are important, considerably less attention has been paid in the literature to some salient features on the supply-side of the markets for care that are instrumental in the implementation of CDMPs. A number of papers (e.g., [5–7] and [17]) have addressed the question of whether the supply-side institutional characteristics of health care settings may be influential. The question of how economic incentives may affect practitioners’ engagement in the optimal management of chronic diseases, such as diabetes, has also been addressed by economists (e.g., [14]).

There is, however, a paucity of literature on whether or not competition between the practitioners who are influential in the application of guidelines—in many cases, medical specialists—plays a role. To the best of our knowledge, though, there is no existing economic literature on the role that inter-professional competition between specialists may play in the design and application of guidelines and/or recommended treatment protocols. This paper begins to address this gap by studying the role that incentives may play for medical specialists who are expected to implement a CDMP for renal (kidney) disease. It considers the potential role that economic incentives may play between the specialties that compete for patients: chiefly, nephrologists and cardiologists, as well as the role that competition between GPs may play in the Italian health care system.

The incentives faced by medical practitioners seems, to us, likely to be an important determinant of the decisions of patients’ agents—i.e., their treating doctors—to encourage their adherence to a treatment regimen recommended under a CDMP. These include the decisions by general practitioners (GPs) to, or not to refer, to specialists; and decisions by specialists, in turn, as to whether to enrol patients in specific programs and/or involve other specialists, from whom they may face competition. The focus of this paper is precisely on whether such supply-side factors are influential in the compliance with guidelines that are promulgated under a CDMP program: in this case, a CDMP for people with chronic kidney disease (CKD).

Despite an extensive literature review, in which we searched for theoretical or empirical economic literature on the role that specialists, specifically, play in the delivery of CDMPs we found no literature of direct relevance. As far as we are aware, there is surprisingly no extant theoretical or empirical work that addresses how specialists control or influence CDMPs. Hence, the most relevant references are to be found in the literature on doctors as “double-agents”. In this literature, doctors are conceived of as agents who serve two principals: patients and funders. We review that literature, considering specifically the role played by the competition between professionals in different specialties, as well as by the utility that doctors may derive from promoting their patients’ health. Important considerations, in this regard, are the roles that institutional factors and competition forces may be expected to play in respect of practitioners’ decisions to follow CDMP guidelines, since these may affect income and other arguments such as professional prestige as well as patients’ health. We then describe the institutional settings and panel data that are used in the paper, followed by a presentation of the empirical results of estimating differences-in-differences models of the CDMP’s implementation, and conducting placebo tests. The final section presents our discussion and conclusion.

### Physicians as double-agents

Modelling clinicians as double-agents—in the sense that they serve two principals—now has a long history in health economics. The seminal contribution of this kind was by Blomqvist [3] where physicians’ decisions are modelled as agents of two main principals: the patient and the insurer/planner.^1^ In this, and similar models, clinicians maximise an objective function that includes considerations of, and trade-offs between, the potentially conflicting objectives of patients and payers. Maximising their own utility, which includes income and other arguments, they are able to take decisions that are at least partly different from those of the two principals, since the circumstances under which clinicians take decisions are affected, at least partly, by hidden information. Thus, according to this version of the dual agency theory, physicians maximise their objective function taking into account the extent of private information to decide on trade-offs in favour of patients—who may switch provider if unhappy with their care—and payers, who may be able to punish providers (e.g., by removing them from a preferred provider network) who deviate from their demands in specific ways. Blomqvist [3] shows that, in a universal healthcare setting where clinicians are paid a fee-for-service, they typically over-provide services, as it is in their financial interest to do so. Hence, under these circumstances, the clinicians’ behaviour is more aligned with patients’ demands than with those of the payer(s). Conversely, when clinicians are paid under capitation arrangements, they tend to “under-provide” services (from the patients’ point of view), thereby aligning their behaviour more strongly with the demands of payers.

A number of studies have developed Blomqvist’s [3] original approach in two respects. First, some studies modify the context and objective function for the clinician by considering further, specific, institutional arrangements and decision contexts. Second, a number of authors have developed the discussion and modelling of how these institutional arrangements (e.g., capitation, FFS) may interact with the competitiveness of the clinical environment.

Allard et al. [1] elaborate on the double-agent model by investigating the case in which physicians not only act as service providers, but also as gate-keepers for specialist services. They show that a fee-for-service (FFS) payment mechanism may lead to a substitution of more costly specialist and diagnostic care services for lower-cost primary care alternatives,while a capitation system may lead to substitution in the opposite direction.

Iverson and Ma [15] present a model in which clinicians are gate-keepers who decide whether or not to refer patients for a diagnostic test. In their setup, referring patients for tests always comes at a cost to the clinician, and the weight given to patients’ benefits increases as the switching costs to move to another clinician get lower (i.e., the lower is the concentration of the local market). Similarly, Godager et al. [12] develop a model where the physician’s decision to refer to specialty care depends not only on the degree of competition, per se, but on the fact that, by avoiding referrals, they can provide more FFS services to the patient themselves.

Schaumans [19] investigates the effects of an increased level of competition between primary care physicians on their prescribing behaviour. The starting point is, again, that physicians might be forced to signal their greater alignment with the patients’ objectives through more “generous” prescribing, when the threat of switching is higher.

Brekke et al. [4] provide further evidence on the role of competition in influencing the primary care physicians’ decisions to provide patients with sick-leave certification. More precisely, in Norway, primary care physicians treat not only their regular (enlisted) patients, but also treat patients in primary care centres where the matching of doctors with patients is, essentially (and on the intensive margin), random. They find that the same physician is significantly more likely to provide sick-leave certificates, *ceteris paribus,* to their own, regular patients than patients encountered in the emergency centres. Finally, two recent papers have investigated questions that are more closely related to the current paper. Kaarboe and Siciliani [16] address the allocative and distributive consequences of various joint payment schemes for GPs and specialists. In this respect, the authors address the issue of how the interaction between GPs and specialists in the implementation of CDMPs is affected. Their focus is on the societal cost-saving and equity-enhancing implications of various financial mechanisms, including practitioners’ adherence to clinical treatment guidelines. Griebenow [13] develops a model where chronic disease patients have either mild or severe disease, and in which primary and specialists work either in a “team” setting or in solo practice. His model produces the conclusion that team care is strictly superior to solo practice only when the difference in the expected treatment costs is relatively larger for the primary physician. When this is not true, solo practice is weakly superior to team care under plausible assumptions.

## The institutional setting and dataset

In this section, we provide a brief description of Italy’s healthcare system as it operates in the Emilia-Romagna region (pop: 4.5 m) of Northern Italy, and a description of the CDMP of interest as well as the source of the dataset used in this paper. The presentation is discursive, and we defer a discussion of the descriptive statistics from the dataset to a subsequent section (see "Descriptive statistics") of the paper.

The Italian National Health Service is a tax-funded, universal, healthcare system [10]. Private supplementary insurance plays a minor role, with only about 15% of the population covered by a private policy (Ferré et al. 2014). Regional governments in Italy make use of the finances provided by a National Health Fund, but are given almost exclusive responsibility for planning and organising all health care services from public healthcare to primary care, and from outpatient specialist care to hospital care, as well as for healthcare related to social care [9, 10, 22]. To pursue their objectives the Regional governments enjoy a large degree of autonomy both at the macro-level (e.g. entry-regulation for providers, pricing of the services, agreements with GPs), and at the micro-level (e.g. organizational and clinical guidelines).

The CDMP of interest in this paper is an initiative called the program “*Prevenzione Insufficienza Renale Progressiva*” (PIRP—*Prevention of Chronic Kidney Disease*) that is designed to improve the clinical management of people with CKD and, ultimately, to slow the progression of disease and improve survival and quality-of-life. Elsewhere (Connelly, Fiorentini and Iommi, [23]) it has been shown that the program has had positive, and causal, effects on improved adherence, delays in disease progression, and survival. The PIRP program, which is endorsed by the Emilia-Romagna Regional Health Authority, is based on a set of clinical guidelines largely inspired by the K/DOQI guidelines of the National Kidney Foundation [18]. These guidelines build upon a staging methodology mainly based on the patients’ glomerular filtrate rate (GFR) which decreases steadily as the disease advances. More precisely, in 2009 the scientific committee of PIRP issued a staging classification that modified the K/DOQI (2002) guidelines on the basis of a NICE (2008) review that identifies the seven disease stages of CKD:

Stage 1: GFR > 90: with signs of renal parenchymal damage

Stage 2: GFR < 89–60: with signs of renal parenchymal damage

Stage 3a: GFR < 59–45: with or without signs of renal parenchymal damage

Stage 3b: GFR < 44–30: with or without signs of renal parenchymal damage

Stage 4: GFR < 29–15: even without signs of renal parenchymal damage

Stage 5: GFR < 15: even without signs of renal parenchymal damage

Stage 6: GFR < 15: already in dialysis

Under the guidelines, GPs are asked to manage CKD patients (who do not have other acute kidney problems) at stages 1 and 2, and also in stage 3a and 3b if the patients are more than 70 years of age, have no diabetes, or do not experience a rapid decrease in the GFR (> 15% in 3 months). GPs are asked to monitor these patients and encourage their adherence to the regimen of medications, tests, and specialist visits recommended by the guidelines, as well as to manage their diet and exercise appropriately. When CKD patients enter stage 3a and 3b without the aforesaid conditions in terms of age, comorbidities or speed of GFR reduction, GPs are required to send them to the nephrologist responsible for the PIRP program in the local area to be enrolled. From that moment onwards, the patient is co-managed by both GPs and PIRP nephrologists with a more intense monitoring and treatment schedule even if the main responsibility for care remains on the GPs, while the nephrologists are to provide specialist visits and supervision. If, and when, the CKD advances to stage 4 or 5, the case management responsibility shifts onto the nephrologists. They are then subject to more intense monitoring and a treatment program that is designed to delay, or avoid, dialysis or transplant. The guidelines foresee that the nephrologists in charge of the PIRP register enrol patients even if at an early or late stage of CKD for monitoring purposes. Moreover, enrolment in PIRP may occur directly in hospital based nephrologist units following a hospitalization for kidney problems. Table 1 summarises the PIRP Enrolment Guidelines by CKD stage.

**Table 1:** PIRP enrolment guidelines

CKD stage(s)	Age	Primary clinical responsibility: GPs or nephrologists?	Enrolment: appropriate or inappropriate?
1–2	Any	GPs	Inappropriate
3a–3b	< 70	GPs and nephrologists	Appropriate
3a–3b	$\ge$ 70	GPs and nephrologists	Inappropriate *unless* comorbid diabetes *or if* rapid decrease in GFR (> 15% over three months)
4	< 70	Nephrologists	Appropriate
4	$\ge$ 70	Nephrologists	Inappropriate *unless* comorbid diabetes *or if* rapid decrease in GFR (> 15% over three months)
5–6	Any	Nephrologists	Inappropriate

Given these rules, the probability of being enrolled in PIRP depends crucially on the decisions of the GPs and of other specialists taking care of the CKD patients to refer patients to the nephrologists in charge of PIRP enrolment following or not the guidelines as well as on the decisions of the nephrologists to actually register the patients balancing the incentives to have a higher professional control over a greater number of patients and to reduce effort. Hence, in the empirical analysis we investigate the main factors that may influence these practitioners’ compliance with the PIRP guidelines, in the multiple principals-agent framework described in "The Institutional setting and dataset".

The dataset we use was provided by the Regional Health Authority of Emilia-Romagna. It includes all individuals (44,686) over the age of 18 years who were hospitalised in Emilia-Romagna of Northern Italy between 1 January 2011 and 31 December 2013, with a CKD diagnosis. For each individual in the sample, quarterly data were extracted between 1 January 2009 and 31 December 2016 for a total of 1,047,628 observations with an average observation period of 23.4 quarters. The variables used in this paper include the individual characteristics of the patients such as age, CKD stage, Charlson Index (a simple count of co-morbidities ranging from 0–11), their Local Health Authority (LHA) of residence, whether and when they are enrolled in PIRP; the characteristics of GPs, including the number of patients under their care; the number and types of specialist visits; and the number and types of hospitalizations. All diagnostic codes that were used to derive the Charlson Index are derived from International Classification of Disease (ICD) codes assigned to each patient during hospitalisation as an inpatient.

### GP and specialist remuneration and incentives

GPs are paid under a capitation system in Italy. For those GPs, remuneration mainly depends on the number of patients registered with a particular GP, so a concern for GPs under competitive pressure is to recruit and maintain an optimal number of patients, taking into account that there is an upper limit—1,500 during the relevant period—set by the NHS regulations. The patients have the right to switch GPs, and we assume that CKD patients will, *ceteris paribus*, apply pressure to GPs to refer them to the care of a specialist doctor (i.e., we assume that patients universally regard specialist care as a good) and because the PIRP program allows patients to receive additional services and drug provision without co-payments. Hence GPs may have a first incentive to refer even early-stage CKD patients to protect their income. In addition, especially if GPs face a convex effort function in the number of patients, there might be a further incentive to refer inappropriately when the number of patients for GP is near to the upper limit.

In order to see if one or both of these incentives are at work, and their relative strength, we create a dummy variable that takes a value of unity if the density—number of GPs over the population, in a given district—is greater than the regional median density, and zero otherwise. A different level of GP density may affect differently the GPs’ decisions (not) to refer to specialists through two different channels. First, since GPs in Italy are paid via capitation, the incentive to refer patients to specialists to reduce GPs’ effort should be stronger in low-density areas where the number of patients for GP is high, due to the convexity of the effort function. On the contrary, the incentives to refer to specialists to retain patients willing to receive specialist treatment, should be stronger in high-density areas where GPs on average have fewer patients and finding other GPs willing to accept additional patients is easier. For these reasons, it is unclear whether GPs operating in areas with a higher (lower) level of density will be more, or less, likely to refer their patients to specialists.

In respect of the incentive structure of the specialists, we hypothesise that the nephrologists have two main incentives to enrol in the PIRP program the CKD patients sent them by GPs, irrespective of their CKD stage. First, nephrologists typically work in hospital units for which an increase in the volume of activities allows to negotiate larger budgets with the LHA managers. Secondly, the enrolment may lead to an increase in the fee-for service payments for the nephrologists’ private practice consultancy activities that are allowed under the NHS regulations. We also hypothesise that some of the nephrologists, who practice in the main hub hospitals—involved in the design and management of the program—may have an additional incentive due to specific benefits (in the form of block grants and reputation) from the operation of the program. At the same time, however, nephrologists may prefer not to enrol to PIRP, even when prescribed, for effort minimization considerations and/or to avoid congestion of their units that would make it difficult to provide services to the kidney patients that cannot be taken care of by GPs or other specialists. These arguments are likely to be stronger in spoke units that do not play a leading role in the management of PIRP, and in outpatient settings where resources are more limited.

In respect of inter-specialist competition, we hypothesise that other specialties involved in the treatment of patients with CKD—especially cardiologists—will be comparatively less likely to refer patients to the nephrologists in charge of PIRP, even when it is appropriate to do so according to the guidelines. This hypothesis is motivated by the observation that cardiologists, *ceteris paribus* in terms of effort considerations, may lose control of patients, when the latter are transferred to the primary care of a nephrologist with the aforesaid negative consequences in terms of hospital unit budgets and fee-for-service payment.

Following the dual-agency model, the planner/payer (i.e., the Local Health Authority) demands that GPs and specialists to comply with the PIRP guidelines to allocate scarce resources only to those patients that have reached a critical point in the progression of CKD. For those patients, the specialist’s intervention and a more intensive monitoring regimen is considered crucial to prolong the time before the patient’s disease requires dialysis. Indeed, imperfect compliance results in (i) an overcrowding of the PIRP program, with patients with immature disease use scarce resources that are meant for the treatment of patients with advanced disease; and (ii) delays in referrals of patients with suitably-advanced disease who would benefit from the more intensive management protocols offered under the program. The planner, through the LHAs, can detect when practitioners refer patients to PIRP deviating from the guidelines. There are no explicit sanctions for non-compliance, but practitioners may be audited to investigate the reasons for deviations from them. Hence, to contrast the individual incentives discussed above, all the practitioners face at least a reputational cost in case of imperfect compliance.

## Empirical strategy

### Differences-in-differences (***DiD***_***M***_) estimation

Our empirical strategy is to model the effect of nephrologist visits on enrolments in the PIRP program. To do so, we adopt a robust estimator developed by de Chaisemartin and D’Haultfoeuille [8] for a continuous treatment, with staggered sharp design. This approach does not rely on the homogenous treatment-effects (TE) assumption required for two-way fixed-effects models (TWFE) and avoids the “negative weights” problem that may seriously bias TE estimates obtained via TWFE. It also enables a direct test of the parallel trends assumption using placebo estimators which compare the outcome evolutions of switchers and non-switchers. Under the latter assumption, the computed placebo estimators should not be statistically significantly different from zero. In addition to these advantages, their estimator also accommodates the modelling of dynamic treatment effects, enabling us to estimate TEs beyond the first “switching” period.

The econometric models we estimate in our main specifications are of the following form:

$${PIRP}_{i,t}={\beta }_{j}{SPEC\_VISITS}_{i,t}^{p}+{\gamma }_{k}{GP\_DENSITY}_{i,t}+{\sigma }_{l}{AGE}_{it}+{\pi }_{m}{SEX}_{i}$$

1 $${+ \alpha }_{i}+{\delta }_{t}+{\varepsilon }_{it}$$

where: ${PIRP}_{it}$ = 1 if person *i* is enrolled in PIRP, in quarter *t*; = 0 otherwise; ${SPEC\_VISITS}_{i,t}^{p}$ = a count of doctor visits to specialist type *p* (e.g., nephrologist, cardiologist), by person *i*, in quarter *t*; ${GPDENSITY}_{it}$= 1 if the general practitioner (GP) market, of person *i*’s GP is above the median level of concentration at time *t*; = 0 otherwise; ${AGE}_{i,t}$ = 1 if person *i* is $\ge$ 70 years of age at quarter *t*; = 0 otherwise; ${SEX}_{i}$ = 1 if person *i* is male; = 0 otherwise; ${\alpha }_{i}$ = an individual fixed-effect; ${\delta }_{t}$ = a time (quarter) fixed-effect; and ${\varepsilon }_{it}$ = a stochastic error term.

We estimate the models reported here using the user-written Stata program *did_multiplegt* developed by de Chaisemartin and D’Haultfoeuille [8].

In our main specification, the specialist visits of interest in (1) are visits to nephrologists. Thus, we use the count of nephrologist visits, by quarter, for each individual, as our “treatment” in the main analyses. Our empirical strategy is to segment the sample by CKD severity in order to determine whether ${\beta }_{j}$ differs by stage, as the treatment guidelines indicate it should, and to test for the incentive compatibility of the guidelines for nephrologists and GPs. Specifically, the guidelines recommend that patients with early CKD (stages 1 and 2) should not be referred to the PIRP program, but should be managed primarily by their GP. Similarly, the treatment guidelines recommend that patients with late CKD (stages 5 and 6) are too advanced in their disease to be referred to the PIRP program and should also not be referred into it. We produce ${\beta }_{j}$ estimates for CKD Stages 3 and 4 separately, as referrals to PIRP may be considered appropriate at both of these stages of disease severity. In each case, we conduct placebo tests for the four quarters (i.e., from *t* − 4 through *t* − 1) preceding a “switch” and produce dynamic estimates for the four quarters following the switch (i.e., for *t* + 1 through *t* + 4).

An important consideration in respect of estimating the ${\beta }_{j}$ in Eq. (1) is how the TEs may be influenced by the age of individuals and the competitiveness of the GP market. In the case of age, the treatment guidelines recommend that, *ceteris paribus*, individuals aged $\ge$ 70 years not be referred to PIRP as the program should slow down the progressive deterioration of the kidney functions to maximize the number of years in good health conditions. In respect of GP density in a local area, we hypothesise (see "The institutional setting and dataset"), that this may influence the recruitment to PIRP due to multiple agency incentives to keep patients and/or to minimize effort. To examine the influence of these factors on the TEs we make a further disaggregation and, for each CKD stage sub-sample, produce estimates for the four combinations of the age and market concentration dummy variables [i.e., (i) high concentration, younger patients; (ii) low concentration, older patients; (iii) high concentration, younger patients; and (iv) high concentration, older patients] and compare them.

#### Placebo tests

We also use the foregoing approach (1) to conduct placebo tests, this time based on a different type of specialist visit. Specifically, in Eq. (1) we replace nephrologist visits with cardiologist visits. Our rationale for these additional placebo tests is that cardiologists do not face direct incentives to refer patients inappropriately in PIRP (i.e., at CKD stages 1–2 or 5–6); although they may face disincentives to enrol (appropriately) patients with CKD stage 3 or 4, in the program, if they view doing so as a threat to their income or professional reputation. In general, though, we should not witness a statistically significant effect of cardiological visits on referrals to PIRP.

### Panel fixed-effects linear probability models (FE LPMs)

The foregoing estimates do not lead to direct tests of several additional hypotheses discussed in the previous sections. Although we control for GP density and age, as well as gender, to produce the *DiD*_*M*_ estimates as outlined above, other hypotheses—such as those concerning the likely behaviour of clinicians working in different types of institutional structure—are not tested explicitly in that framework. Thus, to explore other hypotheses articulated in "The institutional setting and dataset", we estimate additional models with a “standard” fixed-effects (FE) panel, linear probability model (LPM) (FE LPM). The models are estimated on each of the CKD Stage groupings described above, and take the form:

2 $${PIRP}_{it}={\beta }_{0}+{\beta }_{1}{NEPH\_VISIT}_{it}+{\beta }_{2}{CARDIO\_VISIT}_{it} +{\beta }_{3}{OUT\_T}_{it}+{\beta }_{4}{OUT\_HH}_{it}+{\beta }_{5}{GP\_DENSITY}_{it}+{\beta }_{6}{AGE6574}_{it}+{\beta }_{7}{AGE7584}_{it}+{\beta }_{8}{AGE85PL}_{it}+{\beta }_{9}{CKD1}_{it}+{\beta }_{10}{CKD2}_{it}+{\beta }_{11}{CKD3}_{it}+{{\beta }_{12}CKD4}_{it}+{{\beta }_{13}CKD5}_{it}+{{\beta }_{14}CKD6}_{it}+{\beta }_{15}{CKDUS}_{it}+{ \alpha }_{i}+{\delta }_{t}+{\varepsilon }_{it}$$

where the newly-introduced variables are defined as follows: ${NEPH\_VISIT}_{it}$ = 1 if the index nephrologist visit occurs for person *i* in quarter *t*; = 0 otherwise; ${CARDIO\_VISIT}_{it}$ = 1 if the index nephrologist visit occurs for person *i* in quarter *t*; = 0 otherwise; ${OUT\_T}_{it}$ = 1 if person *i* visited a territorial outpatient department in quarter *t*; = 0 otherwise; ${OUT\_HH}_{it}$ =1 if person *i* visited a hospital hub in quarter *t*; = 0 otherwise; ${AGE6574}_{it}$ =1 if person *i* was age 65–74 years in quarter *t* = 0 otherwise; ${AGE7584}_{it}$ =1 if person *i* was age 75–84 years in quarter *t*; = 0 otherwise ${AGE85PL}_{it}$ =1 if person *i* was age 85 years or more in quarter *t*; = 0 otherwise; ${CKD1}_{it}$ =1 if person *i* had CKD stage 1 in quarter *t*; = 0 otherwise; ${CKD2}_{it}$ =1 if person *i* had CKD stage 2 in quarter *t*; = 0 otherwise; ${CKD3}_{it}$ = 1 if person *i* had CKD stage 3 in quarter *t*; = 0 otherwise; ${CKD4}_{it}$ =1 if person *i* had CKD stage 4 in quarter *t*; = 0 otherwise; ${CKD5}_{it}$ =1 if person *i* had CKD stage 5 in quarter *t*; = 0 otherwise; ${CKD6}_{it}$ =1 if person *i* had CKD stage 5 in quarter *t*; = 0 otherwise; ${CKDUS}_{it}$ =1 if person *i* had CKD of uncertain stage in quarter *t*; = 0 otherwise; and all other variables are as previously defined.

## Results

### Descriptive statistics

Since the disaggregation by CKD stage is central in our DiD analyses, we also present the descriptive statistics for the sample according to CKD disease severity. Table 1 shows four panels of descriptive statistics for CKD Stages 1–2 [panel (A)]; CKD stage 3 [panel (B)]; CKD stage 4 [panel (C)]; and CKD stages 5–6 [panel (D)]. There are approximately 17,000 individuals with CKD 1–2 and CKD 3 [panels (A) and (B)]; while the numbers with CKD 4 and CKD 5–6 [panels (C) and (D)] are smaller: approximately 10,000 and 6000 individuals, respectively. The mean age of the sub-samples is generally advanced—in the late 70s—except for CKD stages 5–6, which are the immediate pre-dialytic and dialytic stages of CKD. Specifically, at CKD stages 1–2 the mean age of the population we observe is 78.20 years (SD 12.92), at stage 3 the mean age is 79 (SD 11.99), and at stage 4 the mean age is77.96 (SD 12.53); whereas the mean age is 66.94 (SD 15.00) at stages 5–6. The proportion of males in all sub-samples is approximately 60%, and is slightly larger (64%) for the CKD stages 5–6 subsample. These statistics are coherent with what is known about trends in CKD prevalence and its disproportionate prevalence and worsening, as well as the speed of progression, for males. The clinical complexity of patients – which is proxied by the number of chronic conditions (Charlson index)—increases from 2.13 to 2.42 (+ 15%) from the very early CKD stages (1–2) to the intermediate stages (3–4) and remains stable at later stages.^2^ Hence, the population is made of individuals that are typically multi-morbid from the very early stages of their CKD, and that are likely to require frequent clinical monitoring by more than one specialist (Table 2).

**Table 2:** Descriptive statistics: chronic kidney disease panel data

Variable	CKD stages 1–2					CKD stage 3				
	Variation	Mean	Std. dev.	Min	Max	Variation	Mean	Std. dev.	Min	Max
Gender (= 1 if male; 0 otherwise)	Overall	0.60	0.49	0.00	1.00	Overall	0.61	0.49	0.00	1.00
	Between		0.49	0.00	1.00	Between		0.49	0.00	1.00
	Within		0.00	0.60	0.60	Within		0.00	0.61	0.61
Age (years)	Overall	78.20	12.92	18.00	107.00	Overall	79.00	11.99	18.00	107.00
	Between		11.74	19.80	106.00	Between		11.26	18.67	106.67
	Within		1.22	73.02	83.78	Within		1.15	73.36	84.60
Charlson index (comorbidity count = 0–10)	Overall	2.13	1.54	0.00	10.00	Overall	2.42	1.63	0.00	11.00
	Between		1.50	0.00	9.00	Between		1.58	0.00	11.00
	Within		0.47	− 2.67	6.80	Within		0.50	− 2.98	7.76
GP density (= 1 if concentrated; = 0 otherwise)	Overall	0.57	0.50	0.00	1.00	Overall	0.56	0.50	0.00	1.00
	Between		0.47	0.00	1.00	Between		0.48	0.00	1.00
	Within		0.19	− 0.40	1.53	Within		0.17	− 0.40	1.52
Cumulative nephrologist visits (*N*)	Overall	2.40	12.45	0.00	691.00	Overall	3.30	12.47	0.00	655.00
	Between		12.41	0.00	629.33	Between		12.54	0.00	485.14
	Within		2.78	− 204.13	202.87	Within		3.00	− 428.86	225.14
Cumulative cardiologist visits (*N*)	Overall	4.79	12.17	0.00	243.00	Overall	5.12	12.55	0.00	173.00
	Between		11.77	0.00	204.90	Between		12.31	0.00	170.00
	Within		2.62	− 79.06	81.94	Within		2.79	− 99.79	73.12
Cumulative endocrinologist visits (*N*)	Overall	2.36	6.29	0.00	196.00	Overall	2.88	7.64	0.00	474.00
	Between		5.68	0.00	164.85	Between		6.38	0.00	207.38
	Within		1.77	− 58.35	132.25	Within		2.52	− 184.31	283.69
*Outpatient type dummies* hospital Hub (= 1 if yes; = 0 otherwise)	Overall	0.07	0.26	0.00	1.00	Overall	0.10	0.30	0.00	1.00
	Between		0.20	0.00	1.00	Between		0.23	0.00	1.00
	Within		0.19	− 0.89	1.04	Within		0.20	− 0.86	1.07
Territorial clinic (= 1 if yes; = 0 otherwise)	Overall	0.32	0.56	0.00	2.00	Overall	0.38	0.57	0.00	2.00
	Between		0.40	0.00	2.00	Between		0.41	0.00	2.00
	Within		0.43	− 1.56	2.26	Within		0.43	− 1.50	2.30
PIRP-enrolled (= 1 if yes; = 0 otherwise)	Overall	0.09	0.28	0.00	1.00	Overall	0.14	0.35	0.00	1.00
	Between		0.25	0.00	1.00	Between		0.30	0.00	1.00
	Within		0.10	− 0.88	1.05	Within		0.12	− 0.82	1.10
PIRP_enrol_new	Overall	0.00	0.07	0.00	1.00	Overall	0.01	0.09	0.00	1.00
	Between		0.10	0.00	1.00	Between		0.13	0.00	1.00
	Within		0.05	− 0.50	0.97	Within		0.07	− 0.49	0.97
*N* = 163,255						*N* = 130,502				
*n* = 17,342						*n* = 17,877				
$\overline{T }$= 9.41						$\overline{T }$= 7.30				

Variable	CKD stage 4					CKD stages 5–6				
	Variation	Mean	Std. dev.	Min	Max	Variation	Mean	Std. dev.	Min	Max
Gender (= 1 if male; 0 otherwise)	Overall	0.58	0.49	0.00	1.00	Overall	0.64	0.48	0.00	1.00
	Between		0.49	0.00	1.00	Between		0.48	0.00	1.00
	Within		0.00	0.58	0.58	Within		0.00	0.64	0.64
Age (years)	Overall	77.96	12.53	19.00	106.00	Overall	66.94	15.00	15.00	100.00
	Between		11.83	20.71	105.50	Between		14.40	17.81	100.00
	Within		1.00	72.96	82.76	Within		1.45	61.34	72.40
Charlson index (comorbidity count = 0–10)	Overall	2.49	1.73	0.00	10.00	Overall	2.43	1.75	0.00	10.00
	Between		1.71	0.00	10.00	Between		1.73	0.00	10.00
	Within		0.45	− 2.51	8.03	Within		0.69	− 3.01	7.83
GP density (= 1 if concentrated; = 0 otherwise)	Overall	0.53	0.50	0.00	1.00	Overall	0.55	0.50	0.00	1.00
	Between		0.48	0.00	1.00	Between		0.47	0.00	1.00
	Within		0.16	− 0.43	1.49	Within		0.21	− 0.42	1.52
Cumulative nephrologist visits (*N*)	Overall	8.39	27.93	0.00	773.00	Overall	65.32	118.02	0.00	1,201.00
	Between		27.39	0.00	756.00	Between		80.83	0.00	856.68
	Within		9.61	− 190.09	395.94	Within		62.38	− 482.43	670.81
Cumulative cardiologist visits (*N*)	Overall	4.71	12.31	0.00	177.00	Overall	2.66	7.28	0.00	181.00
	Between		12.45	0.00	167.80	Between		8.56	0.00	151.00
	Within		2.53	− 59.07	59.18	Within		2.29	− 63.81	85.35
Cumulative endocrinologist visits (*N*)	Overall	2.71	5.97	0.00	104.00	Overall	2.71	6.50	0.00	155.00
	Between		6.08	0.00	104.00	Between		6.47	0.00	130.50
	Within		1.27	− 24.04	24.71	Within		2.05	− 38.01	45.63
*Outpatient type dummies* Hospital hub (= 1 if yes; = 0 otherwise)	Overall	0.19	0.39	0.00	1.00	Overall	0.35	0.48	0.00	1.00
	Between		0.31	0.00	1.00	Between		0.39	0.00	1.00
	Within		0.24	− 0.77	1.15	Within		0.28	− 0.62	1.32
Territorial clinic (= 1 if yes; = 0 otherwise)	Overall	0.49	0.57	0.00	2.00	Overall	0.78	0.54	0.00	2.00
	Between		0.46	0.00	2.00	Between		0.46	0.00	2.00
	Within		0.40	− 1.31	2.41	Within		0.35	− 1.13	2.70
PIRP-enrolled (= 1 if yes; = 0 otherwise)	Overall	0.26	0.44	0.00	1.00	Overall	0.06	0.24	0.00	1.00
	Between		0.37	0.00	1.00	Between		0.23	0.00	1.00
	Within		0.15	− 0.70	1.21	Within		0.15	− 0.90	1.03
PIRP-enrolment quarter										
1 = In the quarter of PIRP enrolment	Overall	0.02	0.13	0.00	1.00	Overall	0.00	0.06	0.00	1.00
0 = never in PIRP	Between		0.18	0.00	1.00	Between		0.15	0.00	1.00
Missing = quarters after the first enrolment quarter	Within		0.09	− 0.48	0.97	Within		0.04	− 0.50	0.96
*N* = 54,576						*N* = 61,799				
*n* = 10,180						*n* = 6007				
$\overline{T }$= 5.36						$\overline{T }$ = 10.29				

The *N*, *n* and $\overline{T }$ data reported in this table are correct for all variables at each CKD stage/grouping, with one exception

The exception is the variable PIRP-enrolment quarter, for which these statistics are *N* = 61,799; *n* = 6007 and $\overline{T }$=10.29. These

smaller sample sizes arise as the variable is constructed so that quarters after the first enrolment quarter are “missing”

As for the characteristics of the supply side of health care services, the density of GPs is fairly constant across the CKD sub-samples (except for advanced CKD), while the cumulative number of nephrologist visits increases marginally from CKD stages 1–2 to 3, and very strongly from stages 3 to 4 and then stages 5–6. By contrast, the cumulative number of the other main specialist visits—cardiologist and endocrinologist visits—is stable across CKD stages. The frequency of cardiologist visits is almost double that of nephrologist visits up to CKD Stage 3, but it falls to approximately half of the latter by stage 4, and comprises an even lower fraction of visits at stages 5–6.

Overall, the descriptive statistics in respect of specialist visits suggest that, until CKD Stage 3, cardiologists are most likely to lead the clinical care of these (mostly) multi-morbid patients, while endocrinologists and nephrologists tend more often to play supporting roles. From CKD stage 3 onwards, however, nephrologists become the specialists that are more frequently consulted and are likely to play a greater role in pivotal clinical care decisions for this patient group.

Still focusing on supply side factors, the probability of being hospitalized in a structure that is classified as a hub in the nephrologist network increases continuously as the CKD severity progresses, with a value that goes up from 10 to 19% from stage 3 to stage 4 and up to 35% at stage 5–6. On the other hand, the probability of visiting an outpatient facility outside a hospital goes up more regularly across CKD stages. This outcome seems to be coherent with the strong emphasis of the regional health system on a hub-and-spoke system for the hospital services to concentrate the more complex interventions in relatively less hubs, while outpatient visits—often follow-ups for patients who have been already hospitalised—are offered nearer to the patients’ places of residence.

The probability of being enrolled and treated under the PIRP initiatives increases at all stages, except for the very advanced stages (5–6). The greatest increase in enrolment is again from CKD stage 3 and 4 with a jump of the newly enrolled to 9% and of the treated from 14 to 26%. Overall, the descriptive statistics show that PIRP guidelines suggesting enrolment in PIRP—with a much stronger clinical leadership of the nephrologist at about stage 3—are at least partly being complied with. There are, however, also some deviations from the guidelines with 9% of the CKD stages 1–2 already enrolled in PIRP, and a significant increase in the proportion of enrolled only when patients reach stage 4 and not at stage 3 where it is recommended. Both of these deviations might be related to the incentives of the GPs and of the various specialists involved: the nephrologists might have an interest to anticipate enrolment to follow up patients from an earlier stage, while the other specialists—especially cardiologists who are those more frequently visited during the early stages—might have the opposite interest.

### *DID*_*M*_ results

**Fig. 1:**
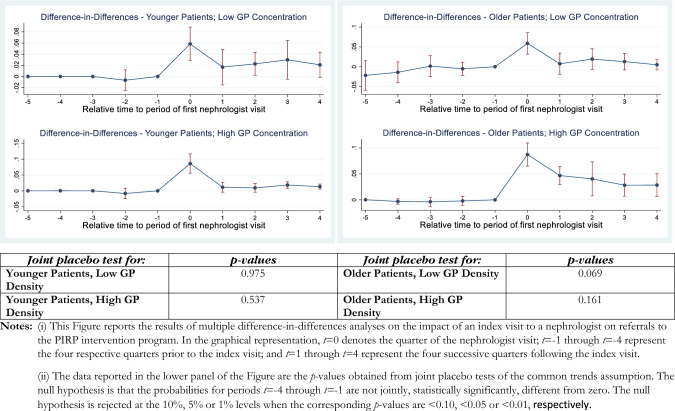
Difference-in-differences estimates of an index nephrologist visit on PIRP program enrolment: CKD stage 3

**Fig. 2:**
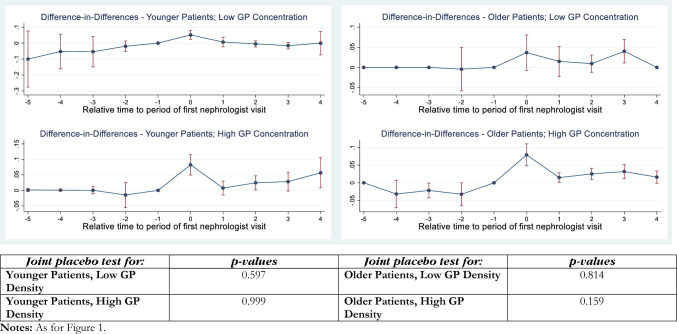
Difference-in-differences estimates of an index nephrologist visit on PIRP program enrolment: CKD stage 4

**Fig. 3:**
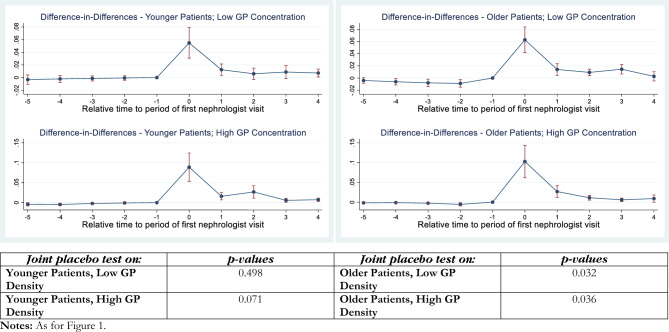
Difference-in-differences estimates of an index nephrologist visit on PIRP program enrolment: CKD stages 1–2

**Fig. 4:**
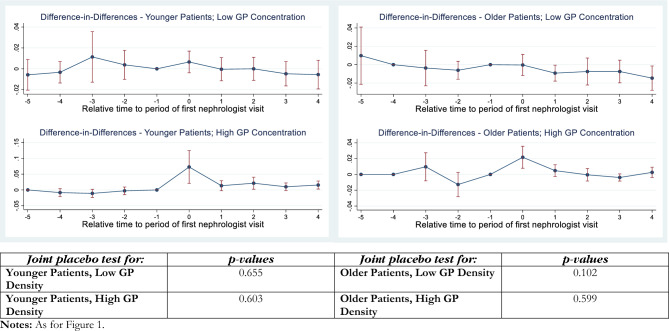
Difference-in-differences estimates of an index nephrologist visit on PIRP program enrolment: CKD stages 5–6

Recall that, in order to examine the different marginal effects of nephrologist visits on the probability of PIRP enrolment, we exploit the fact that clear guidelines are issued for CKD at different stages of severity. We also exploit the fact that the guidelines explicitly state that older patients (ages 70 +) should not be sent to the nephrologists unless there are multi-morbidities or a rapid GFR deterioration, and we combine this with measures of GP density in a given area. To do so, we apply the DID_M_ estimators (i) segmenting by CKD stages; combined with (ii) GP density; and (iii) age dummies. We implement the *DID*_*M*_ estimators using the did_multiplegt developed as a user-written program by de Chaisemartin and D’Haultfoeuille [8] in Stata v.17 (StataCorp 2022). Figures 1, 2, 3 and 4 present our main results (a more complete description of the estimates can be found in Tables S1–S4 of the Supplementary materials).

The interpretation of these Figures is as follows. The panels of each figure show the estimates for the specific CKD stage(s) (e.g., stage 3 in Fig. 1; stage 4 in Fig. 2), particular combinations of the age dummy and GP density combinations. In each instance, moving clockwise from the top left panel, these are: “Younger Patients, Low GP Density”, “Older Patients, Low GP Density”, “Older Patients, High GP Density”, and “Younger Patients, High GP Density”. This layout provides a convenient way of comparing the effects that age (across the figure) and GP density (down the figure) have on the treatment effect, which is the probability that patients are enrolled in PIRP.

The *p *values of “joint placebo tests” of the parallel trends test are reported in the bottom panel of each Figure, in an order that corresponds to their visual representation for the *t* − 4 to *t* − 1 quarters in the panels above. A statistically-significant *p* value indicates a rejection of the null hypothesis that the probabilities of referral for individuals who will remain untreated at *t* = 0 and those who will be treated at *t* = 0 are equal. Setting the critical *p* value at 0.05 (0.10), we reject the null hypothesis of common trends in two (four) instances out of 16 sets of estimates. We will now set the critical value at *p* = 0.05 for the purposes of discussing the results.

Recall that the referral of younger patients (age < 70) to PIRP is generally considered appropriate for patients with stage 3 CKD.^3^ All panels in Fig. 1 show an instantaneous, statistically significant, marginal effect of a nephrologist visit on the probability of enrolment in PIRP, while in three out of four panels the confidence intervals for the estimated dynamic effects of nephrologist visits (i.e., for periods *t* + 1 through *t* + 4 at the top and bottom left panels) mostly encompass at least one zero with the exception of older patients in high-concentration areas. Both instantaneous and dynamic effects do not vary significantly for younger and older patients, while especially the point estimates differ significantly across different levels of GP density. More specifically, the instantaneous enrolment probabilities in GP high-density areas—for both younger and older patients—are higher than in GP low-density areas.

Figure 2 presents the *DID*_*M*_ treatment effect estimates for patients with CKD Stage 4. In each of the four panels there is a positive point estimate of the instantaneous treatment effect, although the confidence interval encompasses zero for older patients in low GP density areas and the dynamic effect estimates are mostly close to zero. Even for CKD stage 4 patients, in areas with high GP density, the instantaneous effects are significantly higher, and the dynamic effects grow slightly over time.

We now turn to the results estimated on CKD stages 1–2 (Fig. 3) which, like CKD stages 5–6, are considered inappropriate for referral to the PIRP program, irrespective of age. In all cases, there is a positive and statistically significant point estimates of the instantaneous treatment effect and—moving clockwise around the panels—evidence of some positive dynamic effects in these patient groups in panels 2, 3 and 4 of the figure. The instantaneous estimates are higher in GP high-density areas while the dynamic effects are similar across ages and level of GP density. Caution is warranted in respect of the result for older patients in low-concentration markets, though, as the parallel trends test is failed at the five per cent level.

Finally, Fig. 4 presents the *DID*_*M*_ estimates for CKD stages 5–6. These results are for patients with late CKD who are considered inappropriate for referral into the PIRP program, regardless of age. Here, the results suggest stricter compliance with the guidelines with smaller positive instantaneous effects only in areas with higher levels of GP density. The dynamic effects following *t* = 0 are never statistically significant.

#### Placebo test results

The results of our placebo tests (Figures S1–S4 of the Supplementary materials), in which we replace nephrologist visits with cardiologist visits. Recall that the logic of these placebo tests is that cardiologists should not have self-interested reasons to refer patients—especially to refer them inappropriately—in the PIRP program. We have foreshadowed the possibility that cardiologists may face incentives not to refer eligible patients to the program, depending on the dynamics of the competition they face from other specialties, such as nephrologists. In other words, we expect this placebo test to result in zero instantaneous and dynamic treatment effects, at least for older patients in CKD stage 3–4, and patients of any age at CKD stages 1–2 and 5–6.

The results are reported in Figure S1–S4 in the same order as they were reported in Figs. 1, 2, 3 and 4. Fifteen out of the 16 placebo test results conform to our expectations, with no statistically significant differences from zero in the treatment and dynamic effects. The anomaly is the bottom-right panel of Figure S6 which shows a small positive instantaneous effect, but zero dynamic effects. Thus, overall, these placebo tests provide encouragement that the results reported in the preceding section are not idiosyncratic.

### Panel linear probability models with fixed-effects

Table 3 reports the results of estimating a linear probability fixed-effects model on the panel. Recall that the purpose of estimating models of this kind is to test hypotheses—advanced in the previous sections—that were not amenable to being tested in conjunction with the main results produced with *DID*_*M*_ estimators. In this specification, we excluded all individual-quarters where there were no hospital or outpatient visits in order to test the role of institutional and/or organizational characteristics of the practitioners involved (working in a hub or spoke hospital unit and working in a hospital or in an outpatient facility), while controlling for the index visit to nephrologists, and cardiologists, as well as GP density, patient age, and the CKD stage. In these specifications, the dependent variable is once again a dummy variable = 1 if the patient is firstly enrolled in PIRP and = 0 otherwise. The nephrologist and cardiologist indicator variables are also binary, taking the value of one if the patient saw a nephrologist (cardiologist) in that quarter. The specification includes time and quarter dummies, and standard errors were clustered at the individual (patient) level. The resulting sample size is 10,414 individual patients, who are observed, on average, for approximately 1.2 quarters.

**Table 3:** Effects of institutional affiliation and market structure on referrals into PIRP: fixed-effects panel regression results (all CKD stages)

Variables	Coefficient (robust st. err)	95% confidence interval
Index nephrologist visit? (= 1 if yes; = 0 otherwise)	0.136*** (0.026)	0.084–0.188
Index cardiologist visit? (= 1 if yes; = 0 otherwise)	− 0.003 (0.005)	− 0.013 to 0.008
Outpatient type dummies		
Territorial clinic (= 1 if yes; = 0 otherwise)	− 0.000 (0.011)	− 0.003 to 0.002
Hospital hub (= 1 if yes; = 0 otherwise)	0.010*** (0.002)	0.006–0.015
GP density = 1 if above median number of GP per patient; = 0 otherwise)	0.002* (0.001)	− 0.001 to 0.004
Age dummies (reference category: age < 65)		
Age 65–74 (= 1 if yes; = 0 otherwise)	0.003 (0.025)	− 0.046 to 0.052
Age 75–84 (= 1 if yes; = 0 otherwise)	− 0.016 (0.027)	− 0.068 to 0.037
Age 85 + (= 1 if yes; = 0 otherwise)	− 0.032 (0.029)	− 0.090 to 0.025
CKD stage dummies [ICD-M-10 codes]		
CKD stage 1 [585.1] (= 1 if yes; = 0 otherwise)	0.013* (0.007)	− 0.001 to 0.027
CKD stage 2 [585.2] (= 1 if yes; = 0 otherwise)	0.004 (0.008)	− 0.012 to 0.020
CKD stage 3 [585.3] (= 1 if yes; = 0 otherwise)	0.012 (0.010)	− 0.008 to 0.032
CKD stage 4 [585.4] (= 1 if yes; = 0 otherwise)	0.015 (0.010)	− 0.005 to 0.035
CKD stage 5 [585.5] (= 1 if yes; = 0 otherwise)	0.025 (0.017)	− 0.009 to 0.059
CKD stage 6 [585.6] (= 1 if yes; = 0 otherwise)	− 0.008*** (0.067)	− 0.084 to − 0.050
CKD diagnosis unstaged [585.9] (= 1 if yes; = 0 otherwise)	− 0.017*** (0.022)	− 0.117 to 0.029
Constant	− 0.002 (0.002)	− 0.045 to 0.040
Number of observations	12,412	
Number of individuals	10,414	
*R*^2^		
Overall	0.075	
Within	0.128	
Between	0.069	
Variance composition		
sigma_u	0.207	
sigma_e	0.090	
rho (fraction of variance due to u_i)	0.8412	
*F*(15, 18,053)	14.44	
Prob > *F*	0.000	

***p < 0.01, **p < 0.05, *p < 0.10

Consistent with the results from our *DID*_*M*_ analyses, the coefficient on nephrologist visits is positive and statistically significant at the one per cent level. The coefficient on cardiologist visits is negative, but not statistically significant, which is also generally consistent with the results of our placebo tests. The main variables of interest here are the “Outpatient Type Dummies” which indicate whether the patient was treated in a “Territorial Clinic” or a “Hospital Hub”. The coefficient on the first of these is close to zero and is statistically not significant; while the coefficient on the Hospital Hub dummy is positive and statistically significant at the one per cent level. The coefficient on the GP density dummy is small, but positive, even if it is only statistically significant at the 10% level.

The remaining variables in Table 2 are mostly included to avoid specification error, in the form of omitted variable bias, but they also warrant some brief comments. First, it is noteworthy that none of the age dummies is statistically significant, although the dummies on the 75–84 and 85 + age groups have the expected (negative) sign. This result is broadly consistent with the results of our *DID*_*M*_ analyses, which tended to suggest that age cut-offs do not seem to play a crucial role in nephrologists’ decisions to enrol or nor patients in the PIRP program. The CKD stage dummies are mostly statistically insignificant, although the coefficients for CKD Stage 6 and CKD diagnosis unstaged are statistically significant and negative. The latter two negative coefficients are consistent with the guidelines: (i) CKD patients whose stage is undetermined are generally not to be enrolled precisely because, without a stage diagnosis, they are ineligible; and (ii) patients at stage 6 are already undergoing or near to dialysis and are also not eligible for the program.

## Discussion and conclusion

CDMPs are based on guidelines that are implemented by physicians who interact between themselves—and with the patients—in a closely linked network of relationships. This includes not only primary care professionals such as GPs, but also specialists from various disciplines, because most patients who are enrolled in CDMPs are multimorbid. These practitioners act in an agent-multiple principal framework with conflicting incentives to comply with the guidelines on how patients should be treated at different stages. The resulting implementation problems occur even when all the professionals involved work as employees for the same organization (e.g., specialists in an NHS) or are under exclusive contractual agreements with such organization (most GPs in an NHS), and are subjected to centrally planned monitoring and payment mechanisms.

This paper represents an attempt to study the effect of inter-professional competition on the enrolment of patients with CKD in a CDMP that is designed to slow the progression of disease and improve health outcomes. Our empirical results show that, even if the overall compliance with the guidelines is relatively high, the behaviour of GPs is affected by the level of competition in their areas of activity. Specifically, GPs tend to refer more patients to the specialists in the CDMP at all CKD stages in areas where GPs treat a lower number of patients. This is because the patients’ threat of exit in case of denial of a specialist referral, with the subsequent loss of income, is more credible in areas where more GPs are below the maximum number of patients set by the regulators. This seems to indicate that the income-maximization incentive is comparatively more powerful that the effort-minimization incentive which is stronger when the average number of patients for GP is higher. However, we also find that the probability of being referred to the specialist is, *ceteris paribus*, slightly higher for older patients at all stages, even if this is not generally consistent with the CDMP guidelines. As the complexity of care for older patients is likely to be higher, this result may also point to a role for the incentives of GPs to reduce their burden of care.

As for the specialists involved, we show that *coeteris paribus* the marginal effect of a nephrologist visit on the probability of enrolment in the CDMP is very similar for early CKD stages (when it is inappropriate) and middle CKD stages (when it is appropriate). A possible explanation is that, especially for early CKD patients, the incentives for nephrologists to increase the volume of professional activities to negotiate larger budgets and/or to increase the private practice income in face of a potential competition from other specialists prevail over the incentives to reduce effort. The marginal effect declines only when patients are at much more advanced stages (pre dialytic or dialytic ones) for which the clinical leadership of the nephrologists is no longer in contention. At the same time, when CKD patients are seen by cardiologists—who often act as clinical leaders due to patients’ multimorbidity—an increase in the number of cardiological visits does not affect the probability of enrolment in the CDMP led by the nephrologists even when it is recommended to do so. Overall, these results show that the effectiveness of a CDMP—which depends critically on targeting the right patients at the right time—is influenced by the professional competition between GPs and between the various specialists involved.

In our presentation of the practitioners’ double-agency approach, we considered the possibility that the institutional role and the professional reputation may also motivate the decisions of some practitioners. In our empirical work, we do find that the probability of enrolment in the CDMP is significantly higher when patients are referred to nephrologists working in hospital hubs which are likely to be directly involved in the planning and general management of the program. This result suggests that incentives related to the increase in reputation due to their leadership role in a highly visible CDMP may, in fact, be at work.

The effective implementation of a CDMP crucially depends on the incentive compatibility of its main operational rules (guidelines or clinical pathways) with respect to the objective functions of the physicians involved. The main policy conclusion of the paper is that in the design of such CDMPs the planners should seriously consider how to overcome the problems due to conflicting incentives between the crucial players. This not only includes GPs who, in some respects in their dual role, are expected to act as “gate-keepers”, but also between practitioners across the different medical specialties who may act upon economic and reputational incentives, showing less willingness to cooperate with other medical specialties in the implementation of complex CDMP guidelines.

## Supplementary Information

Below is the link to the electronic supplementary material.

Supplementary file1 (DOCX 240 KB)

## Data Availability

Data on health services consumption of CKD patients were obtained from the Regional administrative datasets. This anonymized dataset covers the health consumption of CKD patients who are residents in Emilia-Romagna between 2009 and 2016 and provides information on patients’ characteristics, hospital services, ambulatory services, general practitioners (GPs) and pharmaceutical consumption. This information is collected every quarter (maximum 32 quarters) and includes 44,686 individuals. The final dataset included 43,241 individuals, with 1,016,594 observations across an average of 13.59 quarters. These data are not publicly available but were made available to the authors, under license, by the Emilia–Romagna Regional Health Authority: access to the data requires the approval of the Agency for Health Care and Social Services, Emilia-Romagna.

## Footnotes

1 Both McGuire (2000) and Chandra et al. (2014) provide reviews of the relevant literature.

2 We checked that the outlier value of 11 on the Charlson Index in panel (B) of the Table is correct and found that 13 patients with CKD Stage 3 did, in fact, have a Charlson Index value of 11. The maximum at any other CKD stage was 10.

3 Technically, only patients at CKD Stage 3b, not Stage 3a, should be referred to PIRP, but this disaggregation within CKD Stage 3 is not available to us in this dataset.

## Declarations

### Compliance with clinical guidelines

The role of incentives and competition between practitioners.
