# Supplementary material for: Compliance with clinical guidelines: the role of incentives and competition between practitioners
Source: Eur J Health Econ. 2025 Apr 28;26(8):1487–502. doi: 10.1007/s10198-025-01784-5 (PMC12572000; doi:10.1007/s10198-025-01784-5)
Supplement: Supplementary file 1 — Supplementary file1 (DOCX 240 KB) [file 10198_2025_1784_MOESM1_ESM.docx]

**SUPPLEMENTARY MATERIALS**

**Table S1 Difference-in-Differences Estimates of an Index Nephrologist Visit on PIRP Program Enrolment: CKD Stage 3**

| **Younger Patients, Low GP Density** | | | | | | **Older Patients, Low GP Density** | | | | |
| --- | --- | --- | --- | --- | --- | --- | --- | --- | --- | --- |
|  | **Periods** | **Effects**  **(SEs)** | **95% CIs** | ***N*** | **Switchers** | **Periods** | **Effects**  **(SEs)** | **95% CIs** | ***N*** | **Switchers** |
| Instantaneous Effect | *t=*0 | 0.059  (0.015) | 0.029  0.088 | 3,761 | 497 | *t=*0 | 0.086  (0.016) | 0.055  0.117 | 33,184 | 1,483 |
| Dynamic Effects | *t=*1 | 0.017  (0.016) | -0.015  0.049 | 2,911 | 334 | *t=*1 | 0.011  (0.008) | -0.044  0.027 | 27,331 | 1,131 |
|  | *t=*2 | 0.023  (0.011) | 0.002  0.043 | 2,400 | 220 | *t=*2 | 0.009  (0.007) | -0.004  0.023 | 22,919 | 840 |
|  | *t=*3 | 0.030  (0.018) | -0.005  0.065 | 1,973 | 167 | *t=*3 | 0.018  (0.005) | 0.008  0.029 | 19,303 | 597 |
|  | *t=*4 | 0.021  (0.012) | -0.002  0.044 | 1,682 | 143 | *t=*4 | 0.013  (0.004) | 0.005  0.021 | 16,618 | 522 |
| Average Effect | (*t=*0-4) | 0.047  (0.011) | 0.026  0.068 | 12,727 | 1,361 | (*t=*0-4) | 0.061  (0.011) | 0.040  0.082 | 119,355 | 4,573 |
| Placebo  Effects | *t=*-1 | -0.007  (0.009) | -0.025  0.012 | 2,676 | 191 | *t=*-1 | -0.008  (0.008) | -0.024  0.008 | 26,952 | 740 |
|  | *t=*-2 | 0.000  (0.000) | 0.000  0.000 | 1,741 | 83 | *t=*-2 | 0.000  (0.000) | 0.000  0.000 | 19,097 | 401 |
|  | *t=*-3 | 0.000  (0.000) | 0.000  0.000 | 1,329 | 56 | *t=*-3 | 0.000  (0.000) | 0.000  0.000 | 14,078 | 257 |
|  | *t=­*-4 | 0.000  (0.000) | 0.000  0.000 | 950 | 38 | *t=­*-4 | 0.000  (0.000) | 0.000  0.000 | 10,152 | 165 |
| **Younger Patients, High GP Density** | | | | | | **Older Patients, High GP Density** | | | | |
|  | **Periods** | **Effects**  **(SEs)** | **95% CIs** | ***N*** | **Switchers** | **Periods** | **Effects**  **(SEs)** | **95% CIs** | ***N*** | **Switchers** |
| Instantaneous Effect | *t=*0 | \| 0.086 \| \| --- \| \| (0.016) \| | 0.055  0.117 | 33,184 | 1,483 | *t=*0 | 0.087  (0.011) | 0.065  0.110 | 39,527 | 1,957 |
| Dynamic Effects | *t=*1 | \| 0.011 \| \| --- \| \| (0.008) \| | -0.004  0.027 | 27,331 | 1,131 | *t=*1 | 0.047  (0.009) | 0.029  0.064 | 32,393 | 1,498 |
|  | *t=*2 | \| 0.009 \| \| --- \| \| (0.007) \| | -0.004  0.023 | 22,919 | 840 | *t=*2 | 0.040  (0.017) | 0.008  0.073 | 27,062 | 1,101 |
|  | *t=*3 | \| 0.018 \| \| --- \| \| (0.005) \| | 0.008 0.029 | 19.303 | 597 |  | 0.028  (0.011) | 0.007  0.050 | 22,752 | 804 |
|  | *t=*4 | \| 0.013 \| \| --- \| \| (0.004) \| | 0.005 0.021 | 16,618 | 522 | *t=*4 | 0.028  (0.011) | 0.007  0.050 | 19,370 | 662 |
| Average Effect | (*t=*0-4) | \| 0.061 \| \| --- \| \| (0.011) \| | 0.040  0.082 | 119,355 | 4,573 | (*t=*0-4) | 0.091  (0.013) | 0.066  0.115 | 141,104 | 6,022 |
| Placebo  Effects | *t=*-1 | \| -0.008 \| \| --- \| \| (0.008) \| | -0.024  0.008 | 26,952 | 740 | *t=*-1 | -0.002  (0.004) | -0.011  0.007 | 31,853 | 951 |
|  | *t=*-2 | 0.000  (0.000) | 0.000  0.000 | 19,097 | 401 | *t=*-2 | -0.004  (0.004) | -0.012  0.005 | 22,516 | 501 |
|  | *t=*-3 | 0.000  (0.000) | 0.000  0.000 | 14,078 | 257 | *t=*-3 | -0.003  (0.003) | -0.008  0.002 | 16,301 | 311 |
|  | *t=­*-4 | 0.000  (0.000) | 0.000  0.000 | 10,152 | 165 | *t=­*-4 | 0.000  (0.000) | 0.000  0.000 | 11,617 | 198 |

**Notes**: (i) this table reports difference-in-differences (DiD) results of the effect of an index nephrologist visit on the enrolment of CKD patients in the PIRP program; (ii) the results reported here correspond to those reported graphically in Figure 1 in the manuscript; (iii) the estimates referred to as “instantaneous” effects occur at the quarter of the index nephrologist visit (*t*=0); (iv) the estimates referred to as dynamic effects report the effect of the index nephrologist visits in the four quarters following the index nephrologist visit (*t=*1 through *t*=4); (v) the estimates referred to as average effects are calculated as the mean of the estimated instantaneous and dynamic effects (i.e., of the index nephrologist visit over quarters *t*=0 through *t*=4).

**Table S2 Difference-in-Differences Estimates of an Index Nephrologist Visit on PIRP Program Enrolment: CKD Stage 4**

| **Younger Patients, Low GP Density** | | | | | | **Older Patients, Low GP Density** | | | | |
| --- | --- | --- | --- | --- | --- | --- | --- | --- | --- | --- |
|  | **Periods** | **Effects** | **95% CI** | ***N*** | **Switchers** | **Periods** | **Effects** | **95% CI** | ***N*** | **Switchers** |
| Instantaneous Effect | *t=*0 | 0.052  (0.015) | 0.022  0.081 | 1,121 | 280 | *t=*0 | 0.083  (0.017) | 0.049  0.116 | 7,933 | 770 |
| Dynamic Effects | *t=*1 | 0.007  (0.016) | -0.024  0.037 | 703 | 152 | *t=*1 | 0.008  (0.011) | -0.015  0.030 | 5,863 | 510 |
|  | *t=*2 | -0.005  (0.010) | -0.024  0.015 | 470 | 95 | *t=*2 | 0.024  (0.012) | 0.002  0.047 | 4,623 | 365 |
|  | *t=*3 | -0.016  (0.011) | -0.036  0.005 | 336 | 64 | *t=*3 | 0.028  (0.016) | -0.002  0.059 | 3,683 | 245 |
|  | *t=*4 | 0.000  (0.038) | -0.074  0.074 | 265 | 54 | *t=*4 | 0.057  (0.025) | 0.008  0.105 | 3,030 | 193 |
| Average Effect | (*t=*0-4) | 0.024  (0.010) | 0.005  0.044 | 2,895 | 645 | (*t=*0-4) | 0.061  (0.013) | 0.035  0.087 | 25,132 | 2,083 |
| Placebo  Effects | *t=*-1 | -0.020  (0.017) | -0.053  0.014 | 527 | 81 | *t=*-1 | -0.015  (0.021) | -0.056  0.026 | 5,631 | 283 |
|  | *t=*-2 | -0.054  (0.049) | -0.149  0.042 | 257 | 34 | *t=*-2 | 0.000  (0.006) | -0.011  0.012 | 3,508 | 118 |
|  | *t=*-3 | -0.053  (0.055) | -0.161  0.056 | 139 | 19 | *t=*-3 | 0.001  (0.001) | -0.002  0.003 | 2,210 | 61 |
|  | *t=­*-4 | -0.100  (0.090) | -0.276  (0.076) | 73 | 10 | *t=­*-4 | 0.001  (0.002) | -0.004  0.006 | 1,393 | 34 |
| **Younger Patients, High GP Density** | | | | | | **Older Patients, High GP Density** | | | | |
|  | **Periods** | **Effects** | **95% CI** | ***N*** | **Switchers** | **Periods** | **Effects** | **95% CI** | ***N*** | **Switchers** |
| Instantaneous Effect | *t=*0 | 0.083  (0.017) | 0.049  0.116 | 7,933 | 770 | *t=*0 | 0.079  (0.016) | 0.048  0.110 | 8,559 | 888 |
| Dynamic Effects | *t=*1 | 0.008  (0.011) | -0.015 0.030 | 5,863 | 510 | *t=*1 | 0.015  (0.007) | 0.001  0.028 | 6,284 | 555 |
|  | *t=*2 | 0.024  (0.012) | 0.002 0.047 | 4,623 | 365 | *t=*2 | 0.025  (0.008) | 0.009  0.041 | 4,860 | 348 |
|  | *t=*3 | 0.028  (0.016) | -0.002 0.059 | 3,683 | 245 | *t=*3 | 0.032  (0.010) | 0.012  0.052 | 3,908 | 251 |
|  | *t=*4 | 0.057  (0.025) | 0.008 0.105 | 3,030 | 193 | *t=*4 | 0.016  (0.009) | -0.001  0.034 | 3,198 | 191 |
| Average Effect | (*t=*0-4) | 0.061  (0.013) | 0.035 0.087 | 25,132 | 2,083 | (*t=*0-4) | 0.057  (0.010) | 0.038  0.076 | 26,809 | 2,233 |
| Placebo  Effects | *t=*-1 | -0.015  (0.021) | -0.056 0.026 | 5,631 | 283 | *t=*-1 | -0.032  (0.017) | -0.065  0.000 | 6,043 | 327 |
|  | *t=*-2 | 0.000  (0.006) | -0.011  0.012 | 294 | 23 | *t=*-2 | -0.022  (0.011) | -0.043  -0.001 | 3,773 | 138 |
|  | *t=*-3 | 0.001  (0.001) | -0.002 0.003 | 2,210 | 61 | *t=*-3 | -0.032  (0.020) | -0.071  0.007 | 2,505 | 63 |
|  | *t=­*-4 | 0.001  (0.002) | -0.004  0.006 | 1,393 | 34 | *t=­*-4 | 0.000  (0.000) | 0.000  0.000 | 1,429 | 32 |

**Notes:** (i) as for Table S1; (ii) the results reported in this Table correspond to Figure 2 of the manuscript.

**Table S3 Difference-in-Differences Estimates of an Index Nephrologist Visit on PIRP Program Enrolment: CKD Stages 1-2**

| **Younger Patients, Low GP Density** | | | | | | **Older Patients, Low GP Density** | | | | |
| --- | --- | --- | --- | --- | --- | --- | --- | --- | --- | --- |
|  | **Periods** | **Effects**  **(SEs)** | **95% CIs** | ***N*** | **Switchers** | **Periods** | **Effects**  **(SEs)** | **95% CIs** | ***N*** | **Switchers** |
| Instantaneous Effect | *t=*0 | 0.063  (0.011) | 0.042  0.084 | 45,362 | 2,012 | *t=*0 | 0.089  (0.018) | 0.053  0.124 | 179,540 | 3,723 |
| Dynamic Effects | *t=*1 | 0.014  (0.005) | 0.004  0.024 | 39,601 | 1,592 | *t=*1 | 0.016  (0.005) | 0.007  0.025 | 159,726 | 2,969 |
|  | *t=*2 | 0.009  (0.003) | 0.004  0.015 | 34,426 | 1,347 | *t=*2 | 0.027  (0.008) | 0.011  0.042 | 141,501 | 2,490 |
|  | *t=*3 | 0.014  (0.004) | 0.007  0.022 | 29,599 | 1,076 | *t=*3 | 0.006  (0.003) | 0.001  0.011 | 124,364 | 2,038 |
|  | *t=*4 | 0.003  (0.004) | -0.005  0.010 | 26,108 | 903 | *t=*4 | 0.007  (0.002) | 0.045  0.089 | 109,490 | 1,515 |
| Average Effect | (*t=*0-4) | 0.035  (0.005) | 0.025  0.045 | 175,096 | 6,930 | (*t=*0-4) | 0.067  (0.011) | 0.045  0.089 | 714,621 | 12,735 |
| Placebo  Effects | *t=*-1 | -0.009  (0.003) | -0.015  -0.003 | 39,287 | 1,290 | *t=*-1 | -0.001  (0.011) | -0.003  0.002 | 159,371 | 2,603 |
|  | *t=*-2 | -0.008  (0.003) | -0.014  -0.002 | 29,409 | 851 | *t=*-2 | -0.002  (0.001) | -0.004  (0.000) | 124,299 | 1,811 |
|  | *t=*-3 | -0.006  (0.003) | -0.011  -0.001 | 22,767 | 626 | *t=*-3 | -0.005  (0.002) | -0.008  -0.001 | 96,071 | 1,303 |
|  | *t=­*-4 | -0.004  (0.002) | -0.009  0.001 | 16,863 | 475 | *t=­*-4 | -0.004  (0.002) | -0.008  0.000 | 71,148 | 947 |
| **Younger Patients, High GP Density** | | | | | | **Older Patients, High GP Density** | | | | |
|  | **Periods** | **Effects**  **(SEs)** | **95% CIs** | ***N*** | **Switchers** | **Periods** | **Effects**  **(SEs)** | **95% CIs** | ***N*** | **Switchers** |
| Instantaneous Effect | *t=*0 | 0.063  (0.011) | 0.042  0.084 | 45,362 | 2,012 | *t=*0 | 0.103  (0.021) | 0.062  0.144 | 221,145 | 4,710 |
| Dynamic Effects | *t=*1 | 0.014  (0.005) | 0.004  0.024 | 39,601 | 1,592 | *t=*1 | 0.027  (0.008) | 0.012  0.042 | 196,226 | 3,669 |
|  | *t=*2 | 0.009  (0.003) | 0.004  0.015 | 34,426 | 1,347 | *t=*2 | 0.011  (0.003) | 0.005  0.017 | 173,337 | 3,102 |
|  | *t=*3 | 0.014  (0.004) | 0.007  0.022 | 29,599 | 1,076 | *t=*3 | 0.006  (0.002) | 0.002  0.010 | 151,816 | 2,493 |
|  | *t=*4 | 0.003  (0.004) | -0.005  0.010 | 26,108 | 903 | *t=*4 | 0.009  (0.005) | 0.000  0.018 | 135,091 | 2,194 |
| Average Effect | (*t=*0-4) | 0.035  (0.005) | 0.025  0.045 | 175,096 | 6,930 | (*t=*0-4) | 0.068  (0.012) | 0.045  0.091 | 877,655 | 16,168 |
| Placebo  Effects | *t=*-1 | -0.009  (0.003) | -0.015  -0.003 | 39,287 | 1,290 | *t=*-1 | -0.005  (0.002) | -0.010  -0.001 | 195,878 | 3,314 |
|  | *t=*-2 | -0.008  (0.003) | -0.014  -0.002 | 29,409 | 851 | *t=*-2 | -0.003  (0.001) | -0.004  -0.001 | 151,748 | 2,235 |
|  | *t=*-3 | -0.006  (0.003) | -0.011  -0.001 | 22,767 | 626 | *t=*-3 | -0.001  (0.001) | -0.003  0.000 | 119,379 | 1,669 |
|  | *t=­*-4 | -0.004  (0.002) | -0.009  0.001 | 16,873 | 475 | *t=­*-4 | -0.002  (0.001) | -0.004  0.001 | 90,398 | 1,205 |

**Notes:** (i) as for Table S1; (ii) the results reported in this Table correspond to Figure 3 of the manuscript.

**Table S4 Difference-in-Differences Estimates of an Index Nephrologist Visit on PIRP Program Enrolment: CKD Stages 5-6**

| **Younger Patients, Low GP Density** | | | | | | **Older Patients, Low GP Density** | | | | |
| --- | --- | --- | --- | --- | --- | --- | --- | --- | --- | --- |
|  | **Periods** | **Effects**  **(SEs)** | **95% CIs** | ***N*** | **Switchers** | **Periods** | **Effects**  **(SEs)** | **95% Cis** | ***N*** | **Switchers** |
| Instantaneous Effect | *t=*0 | 0.007  (0.005) | -0.004  0.017 | 2,874 | 762 | *t=*0 | 0.073  (0.027) | 0.021  0.125 | 37,232 | 2,489 |
| Dynamic Effects | *t=*1 | 0.000  (0.006) | -0.011  0.011 | 1,981 | 418 | *t=*1 | 0.013  (0.008) | -0.002  0.029 | 30,226 | 1,885 |
|  | *t=*2 | 0.000  (0.006) | -0.011  0.011 | 1,495 | 276 | *t=*2 | 0.021  (0.010) | 0.002  0.040 | 25,200 | 1,476 |
|  | *t=*3 | -0.005  (0.006) | -0.016  0.007 | 1,171 | 210 | *t=*3 | 0.010  (0.006) | -0.002  0.022 | 21,061 | 1,088 |
|  | *t=*4 | -0.006  (0.007) | -0.019  0.008 | 985 | 181 | *t=*4 | 0.016  (0.007) | 0.003  0.028 | 17,996 | 889 |
| Average Effect | (*t=*0-4) | 0.001  (0.003) | -0.005  0.007 | 8,506 | 1,847 | (*t=*0-4) | 0.039  (0.012) | 0.015  0.062 | 131,685 | 7,827 |
| Placebos | *t=*-1 | 0.004  (0.007) | -0.010  0.018 | 1,711 | 186 | *t=*-1 | -0.003  (0.006) | -0.015  0.009 | 29,467 | 1,120 |
|  | *t=*-2 | 0.011  (0.012) | -0.013  0.036 | 900 | 77 | *t=*-2 | -0.011  (0.007) | -0.024  0.002 | 20,582 | 587 |
|  | *t=*-3 | -0.003  (0.005) | -0.014  0.007 | 597 | 43 | *t=*-3 | -0.008  (0.006) | -0.021  0.004 | 14,983 | 361 |
|  | *t=­*-4 | -0.006  (0.007) | -0.020  0.009 | 334 | 29 | *t=­*-4 | 0.000  (0.001) | -0.001  0.001 | 10,665 | 226 |
| **Younger Patients, High GP Density** | | | | | | **Older Patients, High GP Density** | | | | |
|  | **Periods** | **Effects**  **(SEs)** | **95% CIs** | ***N*** | **Switchers** | **Periods** | **Effects**  **(SEs)** | **95% Cis** | ***N*** | **Switchers** |
| Instantaneous Effect | *t=*0 | 0.000  (0.006) | -0.012  0.011 | 4,363 | 964 | *t=*0 | 0.022  (0.007) | 0.008  0.036 | 4,771 | 997 |
| Dynamic Effects | *t=*1 | -0.009  (0.004) | -0.018  0.000 | 3,401 | 667 | *t=*1 | 0.005  (0.004) | -0.003  0.012 | 3,282 | 476 |
|  | *t=*2 | -0.007  (0.007) | -0.022  0.007 | 2,784 | 492 | *t=*2 | 0.004  (0.004) | -0.008  0.007 | 2,505 | 340 |
|  | *t=*3 | -0.007  (0.006) | -0.020  0.005 | 2,312 | 361 | *t=*3 | -0.004  (0.002 | -0.008  0.001 | 2,022 | 259 |
|  | *t=*4 | -0.015  (0.007) | -0.028  -0.001 | 1,972 | 287 | *t=*4 | 0.003  (0.003) | -0.004  0.009 | 1,725 | 212 |
| Average Effect | (*t=*0-4) | -0.005  (0.003) | -0.012  0.002 | 14,832 | 2,771 | (*t=*0-4) | 0.008  (0.003) | 0.003  0.013 | 14,305 | 2,284 |
| Placebos | *t=*-1 | -0.006  (0.005) | -0.016  0.004 | 2,998 | 290 | *t=*-1 | -0.013  (0.008) | -0.028  0.003 | 2,876 | 242 |
|  | *t=*-2 | -0.004  (0.010) | -0.023  0.016 | 1,953 | 138 | *t=*-2 | 0.010  (0.009) | -0.008  0.027 | 1,495 | 106 |
|  | *t=*-3 | 0.000  (0.000) | 0.000  0.000 | 1,353 | 88 | *t=*-3 | 0.000  (0.000) | 0.000  0.000 | 834 | 64 |
|  | *t=­*-4 | 0.010  (0.016) | -0.021  0.041 | 801 | 61 | *t=­*-4 | 0.000  (0.000) | 0.000  0.000 | 475 | 45 |

**Notes:** (i) as for Table S1; (ii) the results reported in this Table correspond to Figure 4 of the manuscript.

**Figure S1 Placebo Estimates: Difference-in-Differences of Cardiologist Visits on PIRP Program Enrolment: CKD Stage 3**

**Figure S2 Placebo Estimates: Difference-in-Differences of Cardiologist Visits on PIRP Program Enrolment: CKD Stage 4**

**Figure S3 Placebo Estimates: Difference-in-Differences of Cardiologist Visits on PIRP Program Enrolment: CKD Stages 1-2**

**Figure S4 Placebo Estimates: Difference-in-Differences of Cardiologist Visits on PIRP Program Enrolment: CKD Stages 5-6**
